# Alginate Micro-fibers encapsulated with oregano essential oil with improved antibacterial efficacy

**DOI:** 10.1039/d6ra04235j

**Published:** 2026-07-17

**Authors:** Abimbola O. Orisawayi, Lu Hao, Ishrat J. Badruddin, Shivam Tiwari, Maria E. Bashawri, Neha Kumawat, Prabhuraj D. Venkatraman, Jonathan A. Butler, Nicole S. Britten, Krzysztof K. Koziol, Sameer S. Rahatekar

**Affiliations:** a Composites and Advanced Materials Centre, Faculty of Engineering and Applied Sciences, Cranfield University Bedfordshire MK43 0AL UK ao.orisawayi@oaustecth.ed.ng abombola..orisawayi@cranfield.ac.uk s.s.rahatekar@cranfield.ac.uk S.S.Rahatekar@lboro.ac.uk bimboris_t@yahoo.com; b Wolfson School of Mechanical Electrical & Manufacturing Engineering, Loughborough University Leicestershire LE11 3TU UK; c Manchester Fashion Institute, Faculty of Arts and Humanities, Manchester Metropolitan University Cavendish Street Manchester M15 6BH UK; d Department of Life Sciences, Faculty of Science and Engineering, Manchester Metropolitan University Chester Street Manchester M1 5GD UK; e Olusegun Agagu University of Science and Technology (OAUSTECH) Okitipupa Ondo State Nigeria

## Abstract

Functional biopolymers are being extensively studied for applications in various industries to replace synthetic polymers due to their natural origin and biodegradability. Study investigates, alginate natural oregano essential oil (OEO) fibres at varied concentrations using wet-spinning process. The incorporation of oregano essential oil significantly influenced fibre morphology, with fibre diameter decreasing from 139.57 ± 6.81 µm in the control fibres to 108.43 ± 2.77 µm at 2 wt% OEO (*p* < 0.0001). The produced fibres exhibited good uniformity across all formulations, as indicated by their low diameter variability. FTIR peaks and TGA data reveal the presence of OEO with alginate. Mechanical tests revealed that with the addition of OEO, tensile strength and Young's modulus of the alginate fibres significantly increased from 69 MPa and 3.3 GPa to 97 MPa and 6.3 GPa, respectively, at 3 wt% OEO loading. The functional wet spun alginate–OEO fibres exhibit antimicrobial properties, with the largest inhibition zones methicillin-resistant *Staphylococcus aureus* and Listeria monocytogenes, with smaller effects against *Salmonella typhimurium* and *Klebsiella pneumoniae* at 2 wt% OEO. This method provides a simple, manufacturing platform to incorporate OEO into alginate while retaining the mechanical and antibacterial properties of the alginate fibres. Our study suggests that the OEO-loaded alginate fibres is suitable for antimicrobial application.

## Abstract 2

Functional biopolymers are being extensively studied for applications in various industries to replace synthetic polymers due to their natural origin and biodegradability.

## Graphical abstract

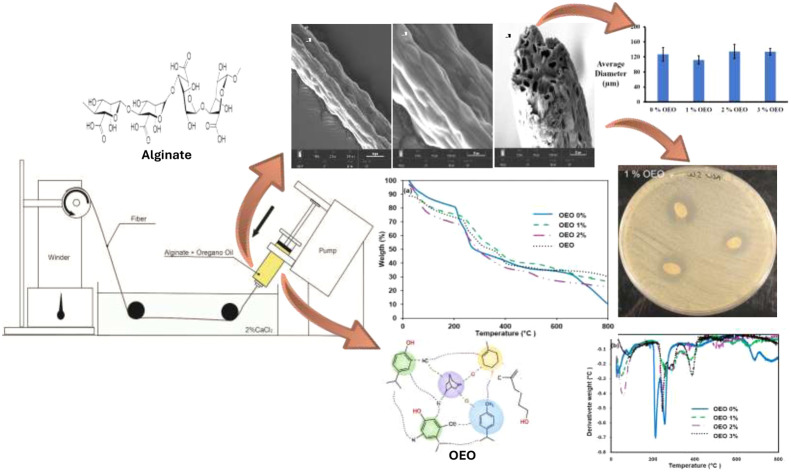

## 1 Introduction

Despite the predominant role of synthetic polymers across various sectors globally, there is a growing movement towards replacing these materials with biodegradable alternatives to reduce environmental impact and dependence on fossil fuel-based polymers.^1,2^ Sustainable biopolymers, including polysaccharides and polyamides, are high molecular weight functional materials derived from natural sources, suitable for a range of high-value applications.^3,4^ Due to their unique properties, these biopolymers are increasingly utilised in the textile and medical fields, alongside their established applications in the food industry.^5–7^ Some biopolymers possess inherent antioxidant and antibacterial properties, while others serve as carriers for these qualities or function as additives.^8–10^ Notably, alginate, chitosan, and cellulose play significant roles in the healing process due to their proliferative, anti-inflammatory, antibacterial, and other targeted actions on specific cells.^9,11^

The bioactive properties of these polymers such as cell proliferation, angiogenesis, antimicrobial effects, and immune modulation foster a microenvironment that is highly conducive to healing.^12^ Alginate is a naturally occurring polysaccharide mainly extracted from various brown algae and marine species.^13^ It is a biocompatible, biodegradable material and is classified as GRAS (generally regarded as safe) algae.^14–17^ Alginate is an anionic, linear polysaccharide consisting of binary copolymers of (1–4)-linked α-l-guluronic (G) and β-d-mannuronic acid (M) residues covalently linked together in different sequences or blocks of consecutive G-residues (G-blocks), consecutive M-residues (M-blocks) or alternating M and G-residues (MG-blocks).^18–20^ Textile surface modification enhances textile features such as antibacterial activity, self-decontamination, hydrophilicity, and biocompatibility while retaining the textile comfort and mechanical strength.^10,21^ In this context, encapsulating essential oils can add antimicrobial activity to biopolymers.

Essential oil (EO) is a concentrated, volatile, hydrophobic oil-like liquid composed of aromatic substances extracted through distillation and solvent extraction of flowers, leaves, roots, bark, fruits, and seeds of herbs that can quickly evaporate and decompose under oxygen, light, and temperature.^22–24^ In this context, encapsulation methods using different biopolymers as wall material may help protect EO, thus allowing controlled release.^25^ Oregano essential oil (OEO) (*Origanum vulgare* L.) is extracted from oregano, comprising mainly terpenes such as thymol, carvacrol, *p*-cymene, γ-terpinene, and linalool, which can break the cell membrane and increase cell permeability.^26^ OEO content is close to the minimum inhibitory concentration (MIC), where the thymol and Carvacrol can disintegrate the outer membrane of *Escherichia coli* (*E. coli*), and *Salmonella typhimurium* (*S. typhimurium*).^27^ In a separate study led by Cui *et al.*, it was reported that OEO can *inhibit Methicillin-resistant Staphylococcus aureus (MRSA)*, by inhibiting the generation of significant pathogenic factor Panton–Valentine leucocidin (PVL) toxin in MRSA.^28^

Use of environmentally friendly, sustainable biopolymers such as alginate has shown great promise as a material for textile antibacterial finishing.^29^ Sobczyk *et al.* developed biopolymer/OEO leaves-based films for potential wound-healing applications and found that a minimum of 1% OEO within alginate films was an effective antimicrobial against Gram-positive bacteria.^30^ Also, on adding OEO, the tensile strength and water vapour permeability decreased with an increase in thickness and elongation percentage.^31^ Encapsulating OEO with alginate can be an environmentally friendly approach towards manufacturing antimicrobial bandages and packaging applications.

The novelty of this study lies in the development of a previously underexplored approach for the fabrication of oregano essential oil (OEO)-encapsulated alginate fibres *via* a fully sustainable wet-spinning process. Although alginate-based fibres and their deposition onto substrates have been reported in the literature, the integration of bioactive essential oil encapsulation within wet-spun alginate matrices remains insufficiently investigated,^10,32^ Previous studies have incorporated oregano essential oil (OEO) into various alginate-based systems, including nanoparticles, hydrogels, films, nano emulsions, microcapsules, coatings, and electrospun nanofibres. These systems have demonstrated promising antibacterial properties; however, most are limited to non-fibrous architectures or electrospun fibre mats with limited mechanical characterisation. As summarised in Table 1, studies investigating OEO-loaded wet-spun alginate microfibres remain scarce. Furthermore, the combined assessment of fibre morphology, mechanical performance, and broad-spectrum antibacterial activity has not been extensively reported for wet-spun alginate/OEO fibre systems.

**Table 1:** Comparative analysis of OEO-incorporated polymer/biopolymer systems and the present study

Number	Study on polymer/biopolymer system	OEO incorporated	Manufacturing method	Quantitative/reported mechanical result	Quantitative/reported antibacterial result	Main limitation compared with present study	References
1	Chitosan–alginate nanoparticles	Yes	Emulsification and electrostatic gelation were used	Not reported as a no fibre was developed in the study	MIC values were 4–32 times lower than free oregano oil against tested bacteria/fungi	Nanoparticles only; no fibre structure as compared with current study	34
2	Alginate/OEO nanofibres	Yes	Electrospinning deposition	Fibre diameter reported as 38–105 nm; micromechanical durability assessed in form of fibre mesh	Tested against MRSA, *L. monocytogenes*, *S. enterica*, and *K. pneumoniae*; OEO improved antimicrobial activity	Electrospun nanofibre mat, fibre were not wet-spun microfibres	35
3	PLA/PCL/OEO/β-cyclodextrin nanofibres	Yes	Electrospinning deposition	OEO/β-CD improved thermal stability but reduced tensile strength	Reported antibacterial/antifungal activity and delayed blackberry spoilage	Not alginate based. But OEO where used	36
4	Sodium alginate/OEO hydrogel membrane	Yes	Solvent casting/crosslinking	Swelling ratio decreased with higher OEO content	Highest OEO membrane showed stronger antioxidant and antibacterial efficacy	Hydrogel, not fibre architecture	37
5	Nanocellulose/OEO film	Yes	Film casting	Film/packaging properties assessed	Tested against *S. aureus* and *E. coli*; oregano and thyme EO films reduced microbial growth in raspberry packaging	Not alginate and not fibre-based	38
6	Sodium alginate nanoemulsion coating	Yes	Nanoemulsion coating	Not a fibre-mechanical study	Evaluated antioxidant and antibacterial activity of sodium alginate nano emulsion coating enriched with OEO	Coating system, not structural fibres	39
7	Alginate–oregano nanofibres on cotton gauze	Yes	Electrospinning/deposition	Electrospun fibre deposition system; mechanical properties not evaluated duet to the nature of the fibre	Developed antibacterial medical bandage from electrospun alginate and oregano nanofibres	Electrospun coating, not wet-spun microfibres	33
8	OEO and active components	Yes	Essential oil antibacterial study	Not applicable	OEO MIC: 0.25–1 mg ml; carvacrol MIC: 0.005–0.04 mg ml^−1^ against Gram-positive and Gram-negative bacteria	No alginate, fibre, or spinning process	40
9	Sodium alginate/OEO microcapsules	Yes	Encapsulation	Not a fibre-mechanical study	Sodium alginate-encapsulated OEO microcapsules developed for antibacterial/antifungal conservation	Microcapsules, not fibres	41
10	Na-alginate/OEO edible film	Yes	Film casting	Limited film-focused evaluation	Tested against *E. coli* O157:H7 and *L. monocytogenes* in Feta cheese storage	Food-packaging film, not fibre	42
	Na-alginate/OEO	Yes	Wet spinning	Fibre diameter: 111.68 ± 11.30 to 134.40 ± 18.70 µm; tensile strength: 69.3 ± 16.9 to 96.7 ± 38.5 MPa; Young's modulus: 3.3 ± 1.0 to 6.3 ± 1.3 GPa	Highest antibacterial activity at 2 wt% OEO; MRSA inhibition zone: 21.7 ± 1.5 mm; activity also observed against *L. monocytogenes*, *S. typhimurium*, and *K. pneumoniae*	Only release kinetics and long-term antibacterial durability not yet investigated	Present study

This work therefore establishes a novel and scalable strategy for producing sustainable, bifunctional alginate-based fibres with enhanced application potential.^33^ The systematic evaluation of OEO encapsulation combined with green reagents on fibre morphology, mechanical performance, and broad-spectrum antibacterial activity remains not extensively explored. Using biodegradable calcium chloride and other environmentally benign reagents, this work establishes a scalable, sustainable, and application-ready approach for antimicrobial fibre-based biopolymers.

The current work focuses on manufacturing encapsulated OEO-based alginate fibres *via* a benign wet-spinning technique and studying their antimicrobial activity, with the hypothesis that oregano essential oil can be successfully encapsulated within wet-spun alginate fibres to form a stable composite structure, where the incorporation of OEO induces concentration-dependent modifications in fibre morphology and intermolecular interactions, leading to improved mechanical properties and enhanced antibacterial activity. It is further hypothesised that there exists an optimal OEO loading at which antimicrobial efficacy is maximised due to a balance between controlled release of active compounds and preservation of fibre structural integrity.

To achieve this, the content of OEO was varied within the alginate solution, and its effect on fibre morphology and mechanical properties was systematically analysed. The manufactured fibres were then subjected to antibacterial testing against methicillin-resistant *Staphylococcus aureus* (MRSA), *Listeria monocytogenes*, *Salmonella enterica* and *Klebsiella pneumoniae*. The results obtained demonstrate the potential of the developed fibres for applications in antibacterial textiles and biomedical materials.

## 2 Materials and methods

### 2.1 Materials

The sodium alginate, oregano (*Origanum vulgare*) essential oil, and calcium chloride (CaCl_2_) used in this study were selected from previous research^33,35^ based on their quality and relevance to ensure experimental robustness. Sodium alginate (alginic acid sodium salt) derived from brown algae. The viscosity is about 5.0–40.0 and of cps *c* = 1%,^17^ ≤15.5%, (*c* = 1%, water@25 °C) loss Drying and (*M̄*_w_) is 161 g mol^−1^ weight-average molecular weight. The ratio of M : (β-d-mannuronic acid to G : (α-l-guluronic acid) (M/G) is 1.61.^43,44^ Product number (Prod. no.): W201502, CAS-number (CAS):9005-38-3 in Table S1 of the S1. The *Oregano vulgare* essential oil (100% purity) was obtained from organic Athina, Greece, its content reported by the manufacturer are carvacrol (80 wt%), thymol (4.2 wt%), *p*-cymol (4.2 wt%), gamma-terpinene (1.6 wt%), limonene (0.22 wt%), sabinen (0.25 wt%), and linalool (0.12 wt%) as the principal components. The calcium chloride (CaCl_2_) us and a crosslinking agent is Anhydrous with granular, ≤7.0 mm, ≥93.0%, *d*: 2.15 g cm^−3^ product of China) with the Prod. no.: 244066, CAS: 10043-52-4 procured from Fisher Scientific agent from Loughborough, UK. The product specifications are detailed in Table S2 of the S2, and pluronic powder (Pluronic^®^ F-127, SIGMA P2443) was obtained from Sigma-Aldrich, UK.

### 2.2 Alginate–OEO solution preparation

An alginate solution (3% w/w) with pluronic (1% w/w) solution was prepared by dissolving in deionised water (DI) at room temperature.^45^ The solution was continuously stirred at 200 rpm for 24 hours at room temperature until complete dissolution, with varying OEO concentrations 0 wt%, 1 wt%, 2 wt%, and 3 wt% added to the alginate solution. The prepared alginate–OEO solution 0 wt%, 1 wt%, 2 wt%, and 3 wt% OEO shown in Table 2 was then transferred to a 60 ml Luer-Lok syringe and placed in the vacuum oven for four hours at room temperature to remove air bubbles.

**Table 2:** Concentrations used to produce alginate fibre with 0%, 1%, 2%, and 3% OEO

Fiber type	Alginate (w/w)	OEO (w/w)	Pluronic (w/w)	CaCl_2_ (w/v)
Alginate–OEO fibers (0 wt%)	3%	0%	1%	2%
Alginate–OEO fibers (1 wt%)	3%	1%	1%	2%
Alginate–OEO fibers (2 wt%)	3%	2%	1%	2%
Alginate–OEO fibers (3 wt%)	3%	3%	1%	2%

**Fig. 1:**
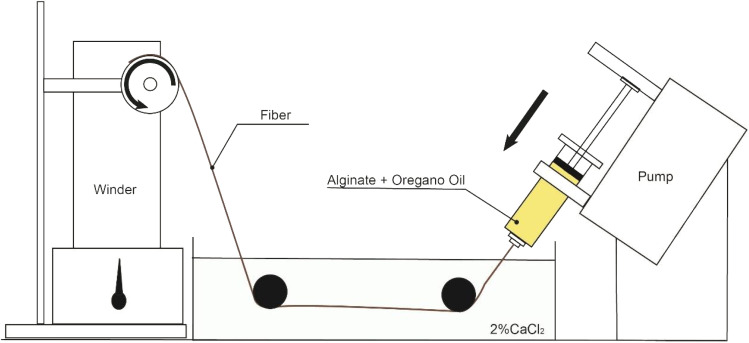
Schematic diagram of the wet spinning process consisting of a pump, a coagulation bath, and a winder for fibre manufacturing.

The equipment required for the fiber-wet spinning process is shown in Fig. 1.^46^ The equipment is mainly divided into three parts: a syringe pump (Nexus 6000) used to pump the solution-loaded syringe at a steady rate, a coagulation tank (made of acrylic) containing three litres of CaCl_2_ (2% w/v) solution for the cross-linking reaction to take place, and a winder used to collect the fibers manufactured after the cross-linking process.

The solution-loaded syringe needle diameter was 0.5 mm, and the needle was immersed in the CaCl_2_ solution to allow the dope solution to cross-link with calcium ions on interaction. The pump speed was set to 0.6 ml min^−1^, and the fibres were collected at 30 rpm. The draw ratio was calculated as the ratio of extrusion velocity to winding velocity and was calculated to be 2.21. Once collected, the produced fibres were further soaked in the CaCl_2_ solution for 15 minutes and air-dried for 24 hours. The alginate–OEO fibres produced were distinguished according to their concentration of OEO in the initial mixed solution and were labelled as 0 wt%, 1 wt%, 2 wt%, and 3 wt% alginate–OEO fibres.

### 2.3 Optical microscope

The produced fibers were investigated using a Leica optical microscope (Nikon Eclipse ME600) and LAS (operating software) to measure the diameter of the produced alginate–OEO fibers. Fibres were analysed using Fiji ImageJ (Version 2.16.0) software to quantify fibre morphological parameters, including length, minimum diameter and maximum diameter. The measured values were exported and statistically analysed using GraphPad Prism version 10.4.1, and histograms were plotted using Microsoft Excel. Data are presented as mean ± standard deviation (SD) based on 25 measurements per group. One-way analysis of variance (ANOVA) was performed to evaluate the effect of oregano essential oil (OEO) concentration on fibre morphology, followed by Tukey's honestly significant difference (HSD) post-hoc test for multiple comparisons.

### 2.4 Scanning electron microscope

A S8000 Environmental Scanning Electron Microscope (ESEM) (TESCAN, Kohoutovice, Czech Republic) was used to scan fine nano-scale images of the electrospun alginate–OEO sample surface. Before SEM imaging, the test samples were coated with AU10 powder to make the sample surface electrically conductive. The samples were analysed using an electron beam of 15–20 keV at room temperature.

### 2.5 FTIR

IR spectra of pristine alginate fibre (0% OEO) and alginate–OEO (2%) were done using a Nicolet iS10 FTIR spectrometer (Thermo Fisher Scientific Co.) under ATR mode with a Ge crystal. The fibre samples were measured with a 400–4000 cm^−1^ scanning range. Each spectrum was collected from 32 scans in the transmission mode.

### 2.6 TGA

Thermogravimetric analysis was performed using the TGA Q500 V20.13 by TA Instruments. The alginate fibres 0%, 1%, 2%, and 3% OEO were weighed, approximately 5 mg of each sample, in a platinum crucible pan. TGA was conducted with an equilibrium at 25 °C, heating at 10.00 °C min^−1^ to 800.00 °C, employing the ramp method under an inert nitrogen gas purge flow of 40 ml min^−1^ and a sample gas flow of 60 ml min^−1^, followed by a 30 minutes air cooling period.

### 2.7 Mechanical test

A tensile test was done using DEBEN 200 newton (N) Microtest Tensile Stage Controller (Suffolk, UK) to measure the tensile strength of the alginate–OEO fibres (0 wt%, 1 wt%, 2 wt%, and 3 wt%). The fibres were held onto a tab of length 10.2 mm, and a force of 5 N was applied. The mechanical properties of the fibres were calculated based on the force, elongation, initial length, and diameter obtained. Young's modulus was calculated based on the stress–strain value obtained.

### 2.8 Antibacterial assays

Alginate–OEO fiber samples were produced in sheet-like form to facilitate the antibacterial assays. The alginate–OEO fibers were coiled into small discs and allowed to air dry at room temperature to form sheet-like samples, as shown in the Supplementary. Antibacterial activity of the alginate–OEO samples was performed using a modified Deutsches Institut für Normung (DIN) 58940–3 standard disc diffusion contact assay method.^47^ Briefly, *Klebsiella pneumoniae* strain 13883, *Listeria monocytogenes* strain Scott A, Methicillin-resistant *Staphylococcus aureus* (MRSA) strain USA300 (ST8) JE2, and *Salmonella enterica* serotype Typhimurium strain 14028 were cultured on Mueller-Hinton (MH) agar (Oxoid, Basingstoke, UK) and incubated at 37 °C for 24 h. Alginate–OEO samples were cut into 6 mm diameter discs and applied to pre-inoculated MH agar plates with bacterial cultures adjusted to a McFarland standard of 0.5. The plates were then incubated at 37 °C under aerobic conditions for 24 h. Bacterial growth zones of inhibition were recorded using a digital calliper gauge. All assays were performed in three biological triplicates.

## 3 Results

### 3.1 Optical microscopy of alginate–OEO wet-spun fibres

**Fig. 2:**
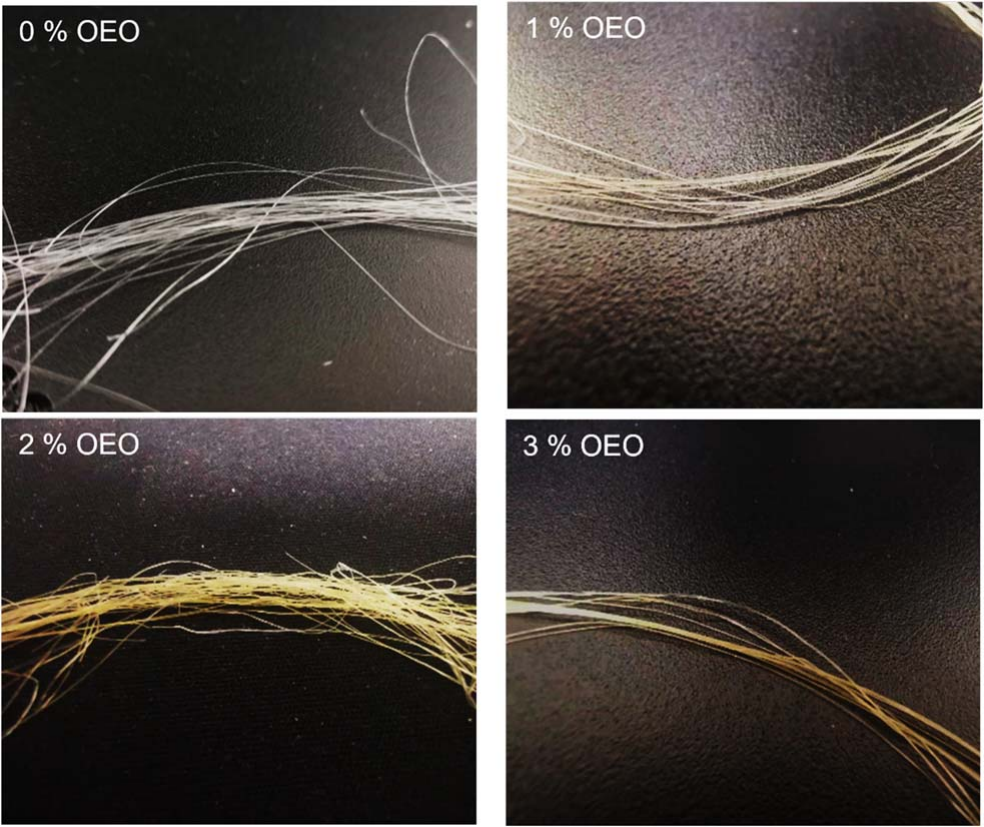
Pictorial representation of the alginate–OEO fibers at 0 wt%, 1 wt%, 2 wt%, and 3 wt% OEO content.

The regular camera images of alginate–OEO (0 wt%, 1 wt%, 2 wt%, and 3 wt%) wet-spun fibres can be seen in Fig. 2. The fibres of pristine alginate with 0% OEO exhibit a white, slightly transparent tint. Images of 1%, 2% and 3% alginate–OEO fibres show that the manufactured fibres became yellow and darker with the increasing OEO content. These results are in line with the optical microscopy images obtained by Andress *et al.*, where the addition of OEO increased the yellow colour of the film with growing OEO concentration (0.25%–0.5%).^30^

**Fig. 3:**
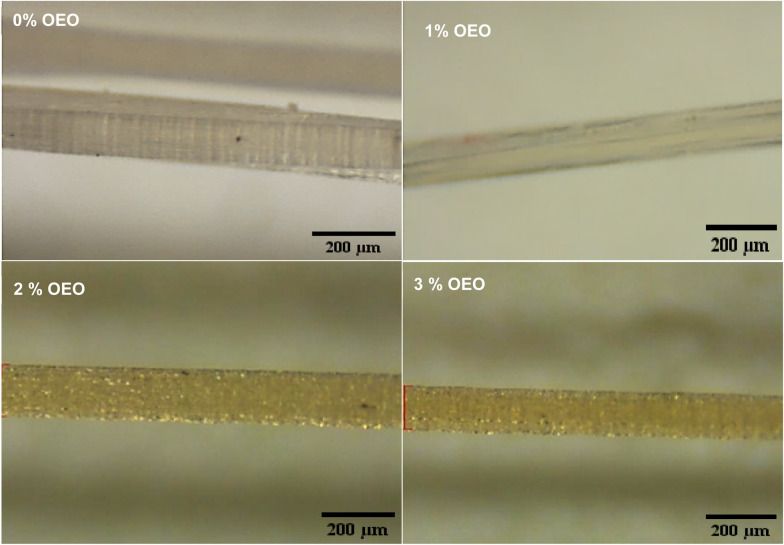
Optical microscopy images of alginate–OEO fibers at 0 wt%, 1 wt%, 2 wt%, and 3 wt% OEO content.

From the optical microscopy images of alginate–OEO (0 wt%, 1 wt%, 2 wt%, and 3 wt%) shown in Fig. 3. The diameters of the alginate–OEO (0 wt%, 1 wt%, 2 wt%, and 3 wt%) fibers were measured. For all four kinds of fibers, 25 different positions of each fiber were taken to measure the mean diameter. The resultant values were calculated to get the mean, minimum and maximum diameter as well as the standard deviation, as shown in Table 3.

**Table 3:** Results of fibre measurements of alginate–OEO nanofibres at different OEO concentrations

OEO content	Mean	Minimum	Maximum	Length
0% OEO	139.57 ± 6.81	122.90 ± 7.89	175.79 ± 11.29	175.75 ± 2.74
1% OEO	142.83 ± 5.42	127.18 ± 9.42	163.28 ± 4.75	126.54 ± 4.49
2% OEO	108.43 ± 2.77	94.75 ± 6.06	136.88 ± 11.07	154.21 ± 3.48
3% OEO	111.82 ± 4.25	90.17 ± 7.67	147.49 ± 19.27	169.86 ± 5.05

**Fig. 4:**
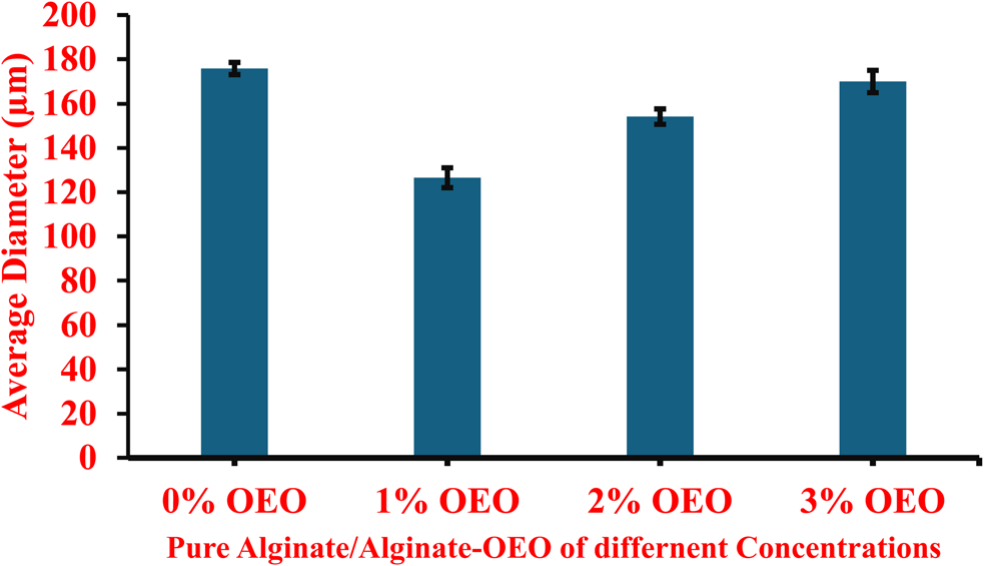
Bar diagram showing the average fibre length of alginate fibres containing 0 wt% OEO (175.75 ± 2.74 µm), 1 wt% OEO (126.54 ± 4.49 µm), 2 wt% OEO (154.21 ± 3.48 µm), and 3 wt% OEO (169.86 ± 5.05 µm). Values are mean ± SD. Significant differences were observed among the groups (*p* < 0.0001).

The diameter of the alginate fibres was influenced by the incorporation of OEO. The average fibre diameter increased slightly from 139.57 ± 6.81 µm for the control fibres to 142.83 ± 5.42 µm at 1 wt% OEO. However, increasing the OEO concentration to 2 wt% resulted in a marked reduction in fibre diameter to 108.43 ± 2.77 µm. A slight increase was subsequently observed at 3 wt% OEO, with a diameter of 111.82 ± 4.25 µm. Overall, fibres containing 2 wt% and 3 wt% OEO exhibited smaller diameters than the control and 1 wt% OEO fibres. The relatively low standard deviation values indicate good fibre uniformity across all samples. Statistical analysis confirmed significant differences in fibre diameter among the groups (*p* < 0.0001). The fibre diameters obtained in this study are comparable to those reported by Zhou *et al.*,^48^ showing a fibre diameter of 136 (±19) µm when spun at a flow rate of 1 ml min^−1^ (Fig. 4).

### 3.2 SEM of alginate and alginate–OEO fibres

**Fig. 5:**
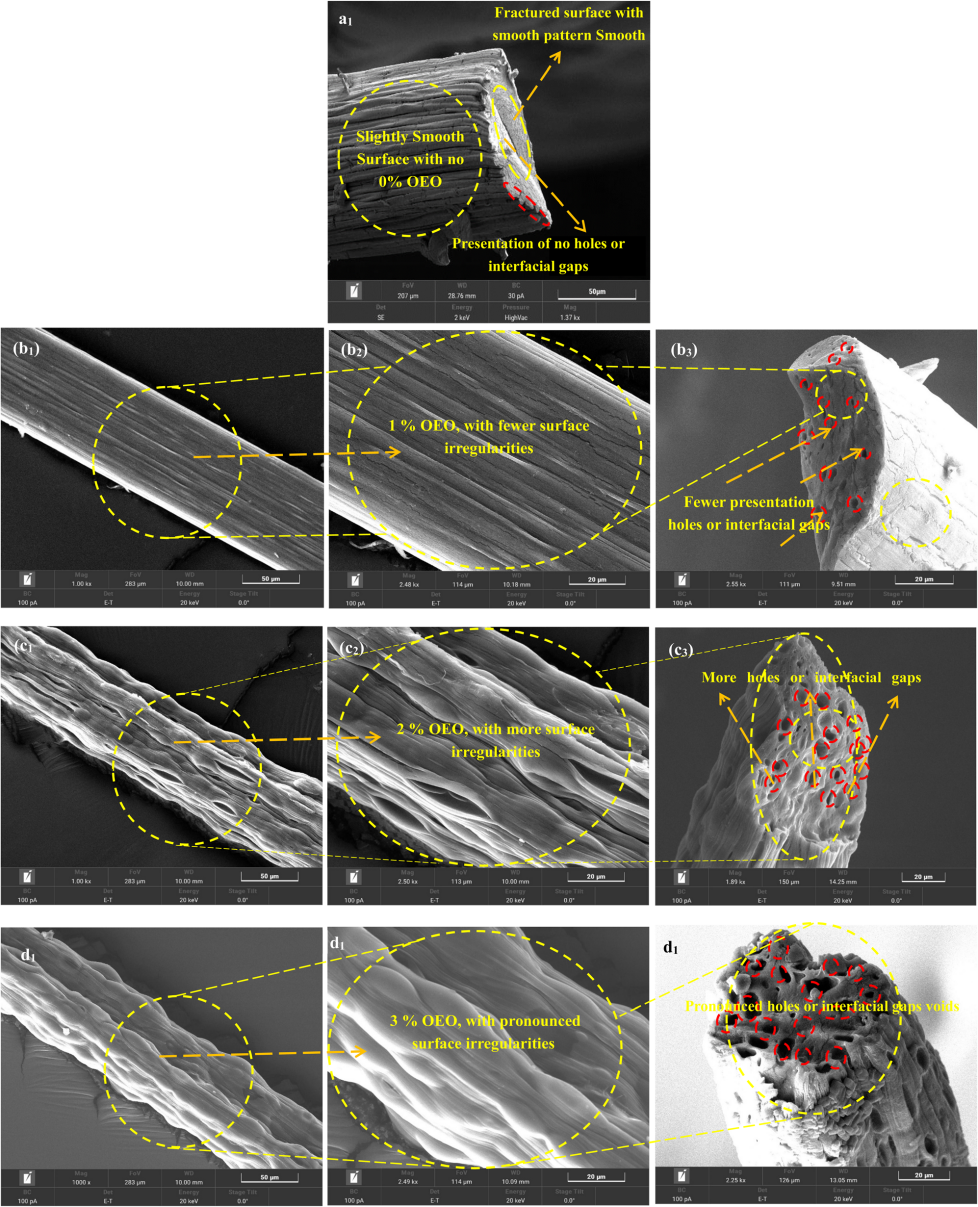
SEM images of the sample's surface morphology and cross sections (a): 0% OEO, (b_1_–b_3_): 1% OEO, (c_1_–c_3_): 2% OEO and (d_1_–d_3_): 3% OEO.

SEM images of pristine alginate fibre and alginate–OEO fibre at varying concentrations are shown in Fig. 5. The surface morphology and the cross-sections of the pure alginate fibre (0% OEO) presented in Fig. 5a, the fibre presents a smooth and clean fracture surface with no observable features such as holes or interfacial gaps, observed in most pure alginate fibres, with similar features to those of pure polysaccharide Wetspun fibres.^49^ Furthermore, the latter looks more features on the surface of the alginate fibre, which looks unique and shrivelled. The deductions from these studies demonstrate the excellent structural integrity of pure alginate fibre.^50^ These characteristics establish a reliable baseline for further material modifications or the incorporation of additives, such as OEO, as the fibre appears homogeneous during the solution preparations, indicating the high quality of the wet-spinning process. Samples^51^

Fig. 5b_1_–d_3_ Further presents fibres with 1%, 2% and 3% OEO as shown, protrusions appear on the fibre surfaces, which become more obvious and larger with increasing OEO content. The fibres appear rougher in the SEM images.^24^ The surface morphology indicates that alginate fibres in Fig. 5b_1_ and 5b_2_ 1% OEO present smoother surfaces, corresponding to the surface of alginate fibres showing a longitudinal alignment typical of wet-spun filaments. The surface is seen as smooth, with minimal ridges and almost no visible microcracks. Surface roughness is seen to be low, indicating that OEO at 1% is well-dispersed and phase separation is minimal. Fig. 5c_1_ and 5c_2_ 2%. These features suggest moderate phase separation of OEO within the matrix, which may enhance diffusion-mediated bioactive release while modestly affecting fibre compactness, and Fig. 5d_1_ and 5d_2_ presents 3% OEO sample that exhibited the highest level of morphological variation, characterised by distinct internal voids and surface roughness resulting from OEO integration with voids inter fibre spaces. Intermediate concentrations of 1% and 2%, as observed from the surface morphology, exhibit a gradual transition in morphology without significant structural distinctions; therefore, the cross-sections were not included to maintain clarity and focus on presenting the most representative differences. The difference is evident in the surface and cross-section of pristine alginate fibre, which shows a very smooth, tight, and solid structure. In contrast, the alginate–OEO fibre (3%) surface and cross-section exhibit many apparent voids inside the fibre and a protruding fibre surface, indicating the presence of OEO. Most of these voids are approximately 3–5 µm in diameter.

In addition, an increase in the concentration of oregano essential oil (OEO) may favour the formation of internal cavities within the alginate-based fibres, primarily due to phase separation, volatilisation, and disruption of the alginate polymer network formation resulting from the addition of excessive essential oils, like previous studies.^52,53^ As a hydrophobic and highly volatile compound, studies show that OEO tends to separate from the hydrophilic alginate matrix during fibre formation, especially at higher concentrations.^54^ This may lead to the entrapment of oil-rich domains that volatilise during drying, resulting in voids or micropores.^54,55^ The presence of more OEO molecules interferes with the tight packing and crosslinking of alginate chains, promoting a less compact fibre structure with increased porosity.^52,53^ The morphology clearly supports this claim, showing the binding of OEO to the alginate fibres while preserving the stable fibre structure. Similar alginate microsphere cross-section due to the presence of essential oil in the alginate microstructures when observed under SEM.^56^ The clusters were causing a structural disorder. This was able to highlight the impact of OEO loading on the alginate matrix, providing valuable insights for optimising composite material properties.

### 3.3 FTIR of alginate–OEO fibres

**Fig. 6:**
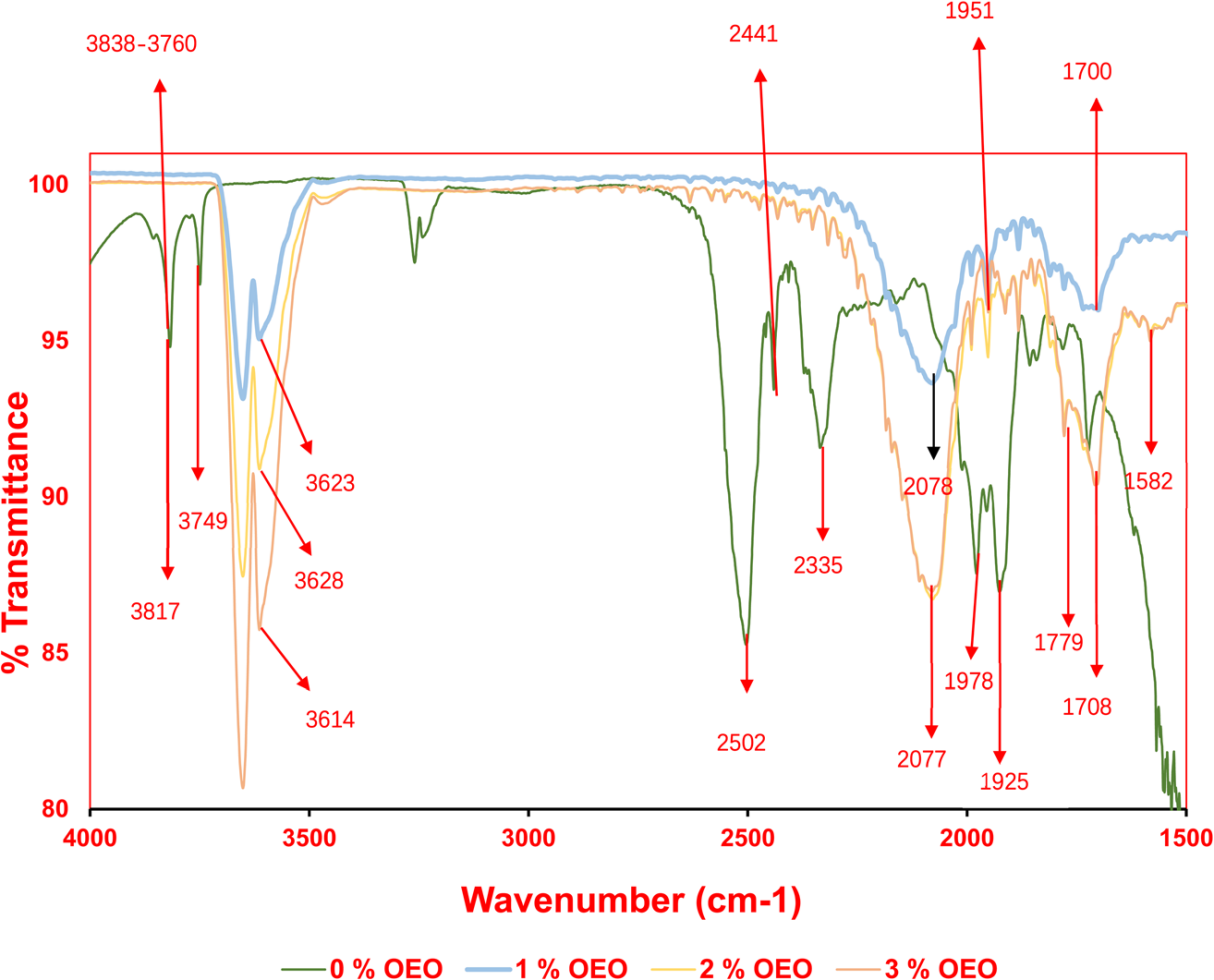
FTIR data of alginate fiber with 0%, 1%, 2%, and 3% OEO.

The FTIR spectrum for alginate fibre with 0%, 1%, 2%, and 3% OEO is shown in Fig. 6. The FTIR spectrum of pristine alginate fibre (0% OEO) showed important absorption bands regarding hydroxyl, ether, and carboxylic functional groups.^57^ The stretching vibration of the O–H bond of 0% OEO alginate fibre shows a pick spectrum in the wavenumber range of 3838 to 3760 centred at 3817 cm^−1^.^58–60^ Other peaks observed at 3749 picks in OEO shifted upon incorporation the OEO shifted slightly from 3614 cm^−1^ to 3623 cm^−1^ and 3628 cm^−1^ for the OEO-loaded fibres, this could be an indication of changes in the hydrogen-bonding environment within the polymer matrix. The alginate loaded OEO fibre also possibly showed a band at 1582 cm^−1^, which shifted towards higher wavenumbers in the OEO-containing fibres, with bands appearing around 1708–1779 cm^−1^. These peaks are associated with the stretching vibration of carboxylate and carbonyl groups^57^

Additional bands were observed at 2502 cm^−1^ and 2441 cm^−1^ in the OEO, suggesting interactions between alginate functional groups and OEO constituents. Peaks detected around 2335 cm^−1^ may be related to atmospheric CO_2_ absorption. The presence and variation of these bands suggest concentration-dependent molecular interactions between alginate and OEO components. The spectral modifications observed at 1582, 1700, 1708, 1779, 1951, and 2077 cm^−1^ confirm the successful incorporation of OEO into the alginate matrix and indicate changes in the chemical environment of the fibres arising from intermolecular interactions between alginate chains and OEO constituents. This interaction mechanism aligns with similar findings by Rahul *et al.* (2025),^61^ who reported the role of hydrogen bonding in stabilising polymer composites and essential oils structures for biomedical applications.^62^ In our study, the alginate matrix acts as a hydrogen bond donor and acceptor, while OEO components, particularly thymol and carvacrol, form secondary interactions *via* their phenolic hydroxyl groups. These interactions contribute to the homogenous encapsulation of OEO droplets within the alginate matrix and improve the fiber structure's internal cohesion, like what has been observed in previous studies.^52^

Overall, the observed peak shifts and changes in band intensity confirm the successful incorporation of OEO into the alginate fibre matrix and indicate molecular interactions between alginate and OEO components. These interactions contribute to the structural modification of the fibre system and support the effective encapsulation of OEO within the alginate network. In conclusion, the observed peak shifts and changes in band intensity confirm the successful incorporation of OEO into the alginate fibre matrix and indicate molecular interactions between alginate and OEO components. These interactions contribute to the structural modification of the fibre system and support the effective encapsulation of OEO within the alginate network.

### 3.4 TGA of alginate–OEO fibres

**Fig. 7:**
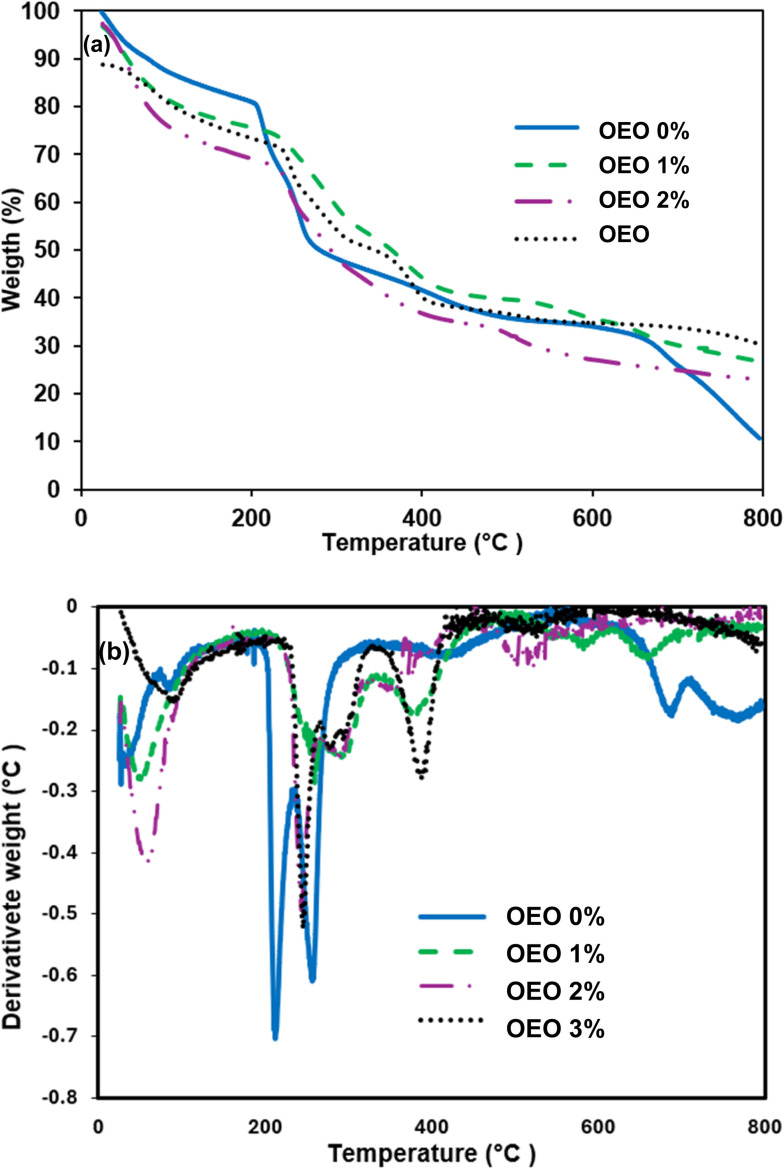
(a) TGA and (b) the DTG curve of samples of 0%, 1%, 2% and 3% OEO fibres.

Fig. 7 presents the thermal profiles of the combined TGA-DTG curves for OEO 0%, OEO 1%, OEO 2%, and OEO 3%, and the curve of the composite with different concentrations. Thermo-gravimetric analysis (TGA) of OEO 0%, OEO 1%, OEO 2%, and OEO 3% fibres shows distinctive properties that align with previous studies. The temperature range 50–210 °C corresponds to the volatilisation of small amounts of free and bound water, and glycosidic bond destruction.^63^

Initial weight loss (moisture evaporation) for pristine alginate and alginate–OEO fibre at 25–200 °C was 23% and 15%, indicating that alginate–OEO fibre has less moisture content than pristine alginate fibre. Pristine alginate fibres, due to their higher stability, showed less mass loss than alginate–OEO fibres at this stage.^64^ This aligns with the result obtained from FTIR data, indicating that OEO forms a bond with the OH groups of alginates, reducing the moisture content. Further destruction of glycosidic bonds was at temperatures 210–440 °C, corresponding to the formation of intermediate material. The thermal weight loss at 250 °C for alginate–OEO fibres was rapid due to evaporative volatilisation of OEO and biopolymer degradation.^65^ The temperature at which 50 % wt loss was observed for pristine alginate fibres and alginate- OEO fibres was 361 °C and 296 °C. Decreasing the temperature to attain 50 % wt loss in alginate–OEO is due to the volatilisation of OEO at a lower temperature than pristine alginate fibres. The process at 440–770 °C is indicative of the oxidation of the previously formed intermediate carbonaceous residue.^66^

### 3.5 Mechanism of OEO constituents within the Ca^2+^ crosslinked alginate network

**Fig. 8:**
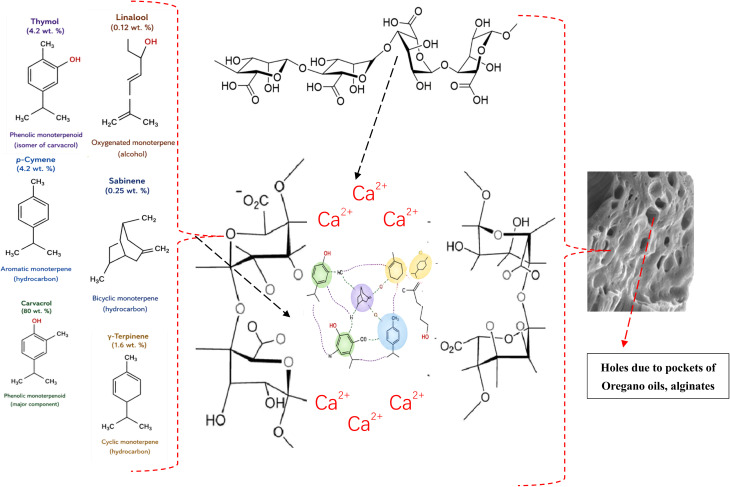
Mechanistic illustration of OEO entrapment in Ca^2+^-crosslinked alginate fibres and the resulting molecular interactions.

The structural schematic (Fig. 8) shows how each OEO constituent interacts with the Ca^2+^-crosslinked alginate network based on its functional groups. Studies shows that carvacrol (80 wt%) and thymol (4.2 wt%), both phenolic monoterpenes, contain an aromatic ring and a phenolic –OH group.^33^ Their structures allow them to form hydrogen bonds with alginate's –OH and –COO^−^ groups, while their hydrophobic rings insert between alginate chains and disturb the uniform Ca^2+^ “egg-box” arrangement. This there is an observation of dual interaction of possibly hydrogen bonding including the hydrophobic insert.^10,32,67^ The schematic representation further explains the main reason these molecules become effectively encapsulated within the fibre network. The non-polar monoterpenes—*p*-cymene (4.2 wt%), γ-terpinene (1.6 wt%), and sabinene (0.25 wt%)—The lack polar functional groups and therefore interact only through hydrophobic embedding within the polymer matrix. Linalool (0.12 wt%), with its single alcohol group, forms weak hydrogen bonds but behaves mostly as a flexible hydrophobic molecule. Their positions in the schematic reflect this: they occupy hydrophobic pockets within the alginate network rather than forming specific interactions which could help in microbial activity.^68–70^

### 3.6 Mechanical properties of alginate–OEO fibers

The data from the tensile test can be combined with the fibre diameter measured earlier to calculate the sample's tensile strength. Four sets for each alginate–OEO fiber (0%, 1%, 2% and 3%) were tested for the mechanical properties as shown in Table 4. This suggests that OEO droplets inside the fibers could reduce their deformability. Table 4 shows the change of ultimate tensile strength and Young's modulus with the increasing content of OEO in the alginate fiber. The average tensile strength of alginate fibers with 0%, 1%, and 2% OEO does not have significant variations, with a range of 60–75 MPa. Their standard deviations are also similar, at around 10–20 MPa. The sample containing 3% Oregano Essential Oil (OEO) demonstrates a higher tensile strength of 96.7 MPa compared to other concentrations. However, this observation does not definitively indicate that the inclusion of OEO enhances the tensile strength of alginate fiber, as the standard deviation associated with the 3% OEO sample is considerably elevated at 38.5 MPa, relative to the other samples. In general, the increase of OEO content will lead to a very uneven distribution of tensile strength of fibers. This results in high variations for the 3% OEO sample in the experimental data above due to the small droplets of OEO in the fibers mentioned above. These small ellipsoidal droplets may locally produce defects in fibers with increasing OEO content, leading to uneven distribution of tensile strength in fibers. Flaws can cause weak points and form cracks during the tensile test for the alginate–OEO fibers, which can lead to breakage.

**Table 4:** Mechanical properties of alginate–OEO (0%, 1%,2%, and 3%) wet spun fibres

OEO content	Ultimate tensile strength (MPa)	Young's modulus (GPa)
0%	69.3 (±16.9)^*p*>0.05^	3.3(±1)^*p*>0.05^
1%	60.8 (±18.4)^*p*>0.05^	3.8 (±1.4)^*p*>0.05^
2%	72.0 (±13.4)^*p*>0.05^	5.7 (±1.3)^*p*>0.05^
3%	96.7 (±38.5)^*p*>0.05^	6.3 (±1.3)^*p*>0.05^

The presence of OEO greatly influenced the tensile strength of the alginate fibers. The enhanced mechanical properties are due to the interaction of OEO with the alginate polymer matrix to form hydrogen bonds. A similar increase in mechanical properties was observed for alginate fibers incorporated with cellulose nano-whiskers (CNW), where, due to the increasing CNW concentration, the tensile strength of the alginate fibers improved from 45.6 MPa to 54.9 MPa, with increasing Young's modulus from 1.58 GPa to 1.79 GPa.^71^ In another study, the tensile strength and Young's modulus of wet-spun sodium alginate/graphene oxide (GO) fibers improved to 0.62 and 4.3 GPa with four wt% GO addition.^72^ The results of our alginate–OEO fibers are better than the mechanical properties obtained for alginate–OEO based films, where the addition of OEO reduced the tensile strength of the alginate films from 71 MPa to a maximum tensile strength of 55.5 MPa on the addition of 0.5 (% p/v) OEO.^31^ Tensile strength (7.7 MPa) and Young's modulus (0.006 GPa) of alginate/chitosan-based film with 0.5% OEO were significantly lower than those of our manufactured alginate–OEO wet spun fibers, showing improved mechanical properties of fibers over films.^30^

### 3.7 Antibacterial activity of alginate–OEO fibers

**Fig. 9:**
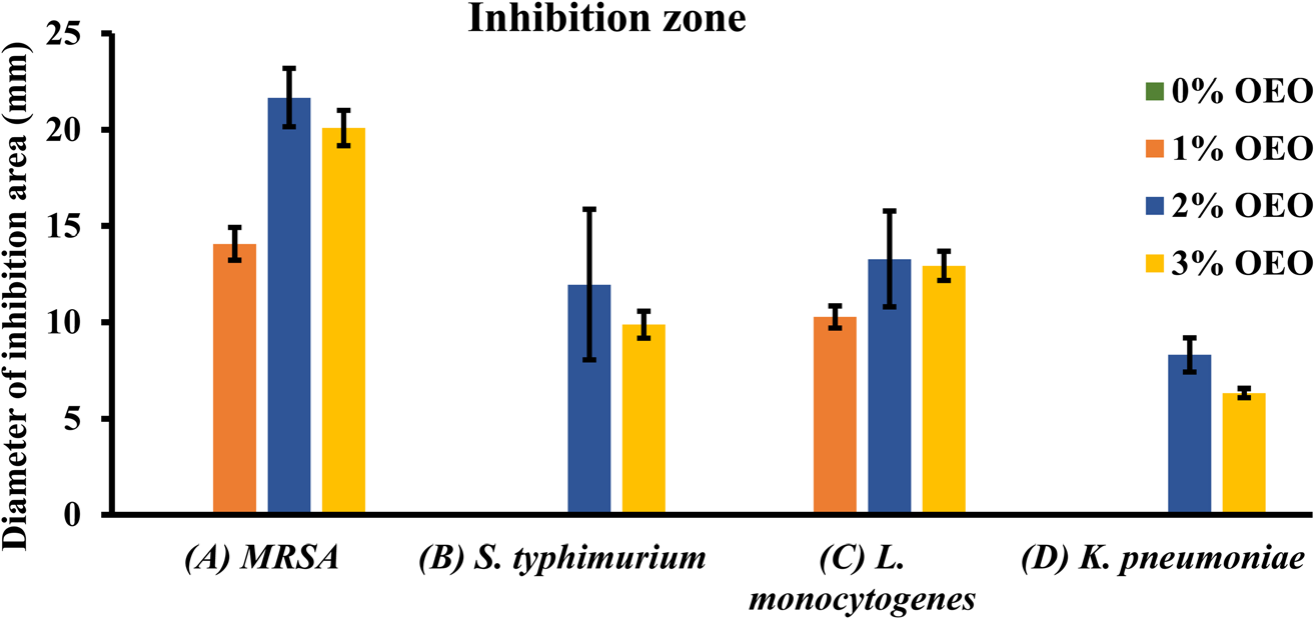
Antibacterial zones of inhibition mediated by alginate–OEO with different concentrations (0% to 3%) against (A) MRSA, (B) *S. typhimurium* (C) *L. monocytogenes*, and (D) *K. pneumoniae*.

The alginate–OEO fibers were made into sheet samples after a drying treatment and were then cut into 6 mm diameter circular discs for use in antibacterial contact assays against *K. pneumoniae*, *L. monocytogenes*, MRSA, and S. *typhimurium*. Alginate–OEO fibers demonstrated antibacterial activity at all concentrations applied against both Gram-positive MRSA and *L. monocytogenes* bacteria (8A and B), whereas only concentrations of 2% and above were active against Gram-negative *S. typhimurium* and *K. pneumoniae* (Fig. 9C and D), with no activity observed at 1%. Alginate–OEO at 2% demonstrated the highest overall antibacterial activity of all concentrations, with zones ranging in mean diameter between 21.7 (±1.5) *p* < 0.05 mm for MRSA (Fig. 9A) to 8.3 (±0.89) *p* < 0.05 mm for *K. pneumoniae* (Fig. 9D). Although a higher OEO loading might be expected to enhance antibacterial performance, the 3% OEO fibres exhibited slightly reduced inhibition zones. This behaviour may be attributed to the non-uniform distribution and possible agglomeration of OEO within the fibre matrix at higher concentrations, which can reduce the effective release and diffusion of active antimicrobial compounds into the surrounding medium. Furthermore, excessive OEO loading may alter fibre morphology and increase encapsulation within the alginate structure, thereby limiting the availability of bioactive constituents responsible for bacterial inhibition. Similar observations have been reported in essential oil-loaded polymeric systems, where an optimum loading concentration provides maximum antimicrobial efficacy, while further increases may negatively affect the release profile of the active compounds. The images of MRSA are used as an example, and the results of the antibacterial test.

**Fig. 10:**
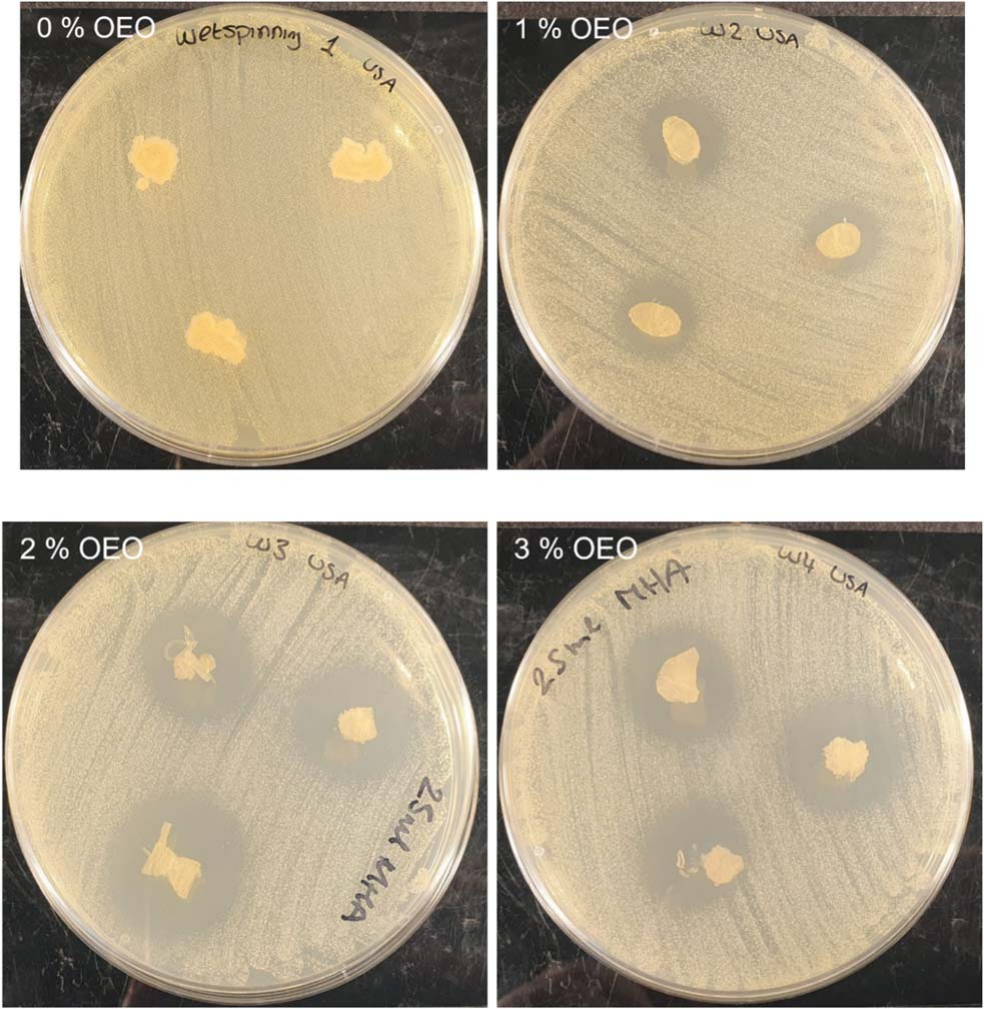
Agar disc-diffusion test of discs of alginate/OEO fibers against *MRSA*.

Fig. 10 shows the images, compared with the sample without OEO, the samples with OEO exhibited pronounced inhibition areas around the samples. Therefore, the alginate–OEO wet-spun fibres strongly inhibited *MRSA*. There was a general decreasing trend in activity between 2% and 3% alginate–OEO, which was significant (*p* > 0.05). However, this demonstrated the OEO releasing characteristics of the alginate, where increasing the concentration did not necessarily correlate with increasing antibacterial activity. No antibacterial activity was observed with alginate alone (0% OEO).

## 4 Discussion

Synthetic polymers have been the backbone of various industries due to their versatility, durability, and cost-effectiveness.^2^ However, their environmental impact, in terms of manufacturing from non-renewable fossil fuels and non-biodegradability is a growing concern. Alginate, due to its abundance in nature, which does not require arable land and freshwater, offers a great advantage over synthetic polymers, and other abundant biopolymers such as cellulose and chitosan.^14,15^ Alginate fibers could be further functionalised by encapsulating oregano essential oil (OEO) within its structure, to enhance its antimicrobial activity for its application across various sectors such as biomedical, antimicrobial textiles, and food-packaging.^73–75^

Previously, OEO has been encapsulated in poly (vinylidene fluoride) polymer (PVDF) through electrospinning for its effect against cancerous cells.^76^ However, the synthetic nature and limited biodegradability of PVDF restrict its applications. Similarly, nanofibers of cellulose acetate encapsulating rosemary and OEO using electro-spinning were made.^77^ It is well known that cellulose is insoluble in common solvents, and usually utilises expensive solvents for its dissolution, thereby increasing the manufacturing cost of the fibers. OEO was encapsulated into chitosan-alginate nanoparticles by emulsification and subsequent electrostatic gelation, exhibiting antimicrobial activity.^78^ The wound dressings fabricated by polymers and oregano essential oil (OEO) can be very effective as hydrogels. OEO encapsulated alginate hydrogels were produced by the solvent casting method, showing antibacterial activity.^79^ Numerous studies have been conducted on encapsulating OEO using the electro-spinning process.^80–82^ However, limited work has been shown to encapsulate and study the effect of OEO in alginate fibers. When comparing the effect of alginate–OEO nanofibers with our manufactured alginate–OEO fibers, the zone of inhibition of fibers is significantly greater than the zone of inhibition of nanofibers, showing a greater inhibitory effect of fibers over nanofibers for similar OEO composition.

This paper focuses on manufacturing alginate and alginate–OEO fibers using a wet-spinning process. It shows the inhibitory effect of varying OEO concentration in alginate–OEO fiber against four selected bacteria (MRSA, *L. monocytogenes*, S. *typhimurium*, and *K. pneumoniae*). The characterisation results show that OEO can be encapsulated into alginate and spun into micron-scale composite fibers *via* wet spinning. The diameters of the fibers ranged from 100–150 µm; for samples containing different concentrations of OEO, insignificant fiber diameter change with increasing OEO content was observed. The microscopy and SEM imaging show the presence of OEO stored as small droplets within the alginate fibers. With increasing OEO content, the droplet-like protrusions visible on the alginate–OEO fiber surface are more noticeable, and the distribution density is also greater. FTIR peaks confirm the bonding of OEO with alginate, which is further supported by the TGA data indicating 23% and 15% initial weight loss for pristine alginate and alginate–OEO fiber at 25–200 °C. Wet-spinning involves extruding dope solution into a coagulation bath comprising divalent cation solution to produce water-insoluble alginate composite fibers. The stretching action during the manufacturing process increases the degree of molecular alignment, thereby improving the overall fiber strength, which is essential for various healthcare applications.^83,84^ No significant change in the alginate–OEO fiber samples' tensile strength (60–75 MPa) was observed for OEO content ranging between 0% and 2%. However, further increase of OEO content to 3% increases the tensile strength of the fibers (96.7 MPa).

Antibacterial assays showed that alginate–OEO demonstrated inhibitory effects against all four selected bacteria. At 1% OEO, inhibitory activity was observed against MRSA and *L. monocytogenes*, with no activity observed against *S. Typhimurium* and *K. pneumoniae*. Both MRSA and *L. monocytogenes* are Gram-positive bacteria which lack an outer membrane and are likely more susceptible to the antibacterial effects of OEO compared to Gram-negative *S. Typhimurium* and *K. pneumoniae*.^85^ However, activity was observed at >2% OEO against both bacterial types, albeit with *MRSA* and *L. monocytogenes* being notably more susceptible, as demonstrated by observing larger inhibitory zones upon exposure. In the present study, increasing the concentration of OEO beyond 2% did not correlate with any further increase in antibacterial activity, suggesting that either there is a limit to the amount of OEO that can be released from the alginate–OEO fibers or there is a diffusion/saturation limit of OEO into the agar over the 24 h assay duration. Further understanding the release characteristics will permit optimisation for future applications.

## 5 Limitations and future perspectives

The findings from this study are promising; however, certain limitations should be acknowledged to provide a comprehensive evaluation of the developed fibres and to guide future research. The antibacterial activity was evaluated only using the agar disk diffusion method, and quantitative antimicrobial assessments such as minimum inhibitory concentration (MIC) and minimum bactericidal concentration (MBC) were not performed. Furthermore, the molecular mechanisms of oregano essential oil (OEO) antibacterial action and its release kinetics from the fibres were not investigated. Future studies should incorporate quantitative antimicrobial analyses and mechanistic investigations to provide a more comprehensive understanding of the antibacterial performance of the developed materials. The optimisation of wet-spinning parameters, such as polymer concentration, coagulation bath composition, extrusion rate, and ambient conditions, was not fully explored, which could improve fibre uniformity and structural integrity. Characterisation techniques, including AFM, XRD, and NMR, were not comprehensively applied, limiting detailed insight into fibre morphology and composition. Future work will focus on optimising wet-spinning conditions, conducting detailed structural characterisation, evaluating OEO release behaviour, and performing *in vivo* studies to validate potential applications in real-world scenarios.

## 6 Conclusion

The use of alginate wet-spun fibres has gained interest due to their ease of handling, manufacturing, and mechanical integrity retention while in the wet state. Oregano, essential oil (0 wt%, 1 wt%, 2 wt%, and 3 wt%) based alginate fibres were manufactured using the wet-spinning process. The manufactured alginate–OEO fibres exhibit significantly improved tensile strength and Young's modulus compared to pristine alginate fibers. This enhancement may be attributed to the interfacial interactions between oregano essential oil (OEO) constituents and the alginate polymer network, where hydrophobic bioactive compounds potentially induce molecular rearrangement, restrict polymer chain mobility, and promote a denser fibre microstructure through improved intermolecular packing and secondary interactions within the biopolymeric matrix. Notably, alginate–OEO at 2% demonstrates the highest overall antibacterial activity, showing superior inhibitory effects against MRSA and L. monocytogenes than against *S. typhimurium* and *K. pneumoniae*. These findings indicate that alginate–OEO fibres are a promising and sustainable functional material. They offer excellent antibacterial efficacy and strong potential for applications in antibacterial textiles and food packaging due to their enhanced mechanical properties. It is important to note that these alginate nano-fibres are water-soluble, restricting their use for packaging items with high water content.

## Author contributions

Abimbola O. Orisawayi, Lu Hao, and Ishrat J. Badruddin: formal analysis, conceptualisation, investigation, methodology, funding acquisition, project administration, software, visualisation, writing the original draft, and writing – review & editing. Shivam Tiwari, Maria Emad Bashawri, Neha Kumawat, Prabhuraj D. Venkatraman, Jonathan A. Butler, Nicole S. Britten: software, visualisation, writing the original draft, and writing, review & editing. Krzysztof K. Koziol, and Sameer S. Rahatekar: supervision, visualisation, writing – review & editing, supervision, and resources.

## Conflicts of interest

The authors declare that they have no known competing financial interests or personal relationships that could have appeared to influence the work reported in this paper.

## Supplementary Material

RA-OLF-D6RA04235J-s001

## Data Availability

The data and supporting documents are available from the corresponding author upon reasonable request. Supplementary information (SI): Tables S1 and S2, which provide the product specifications of the chemicals used in this study, specifically sodium alginate and calcium chloride. Table S1 contains the specifications of sodium alginate, while Table S2 contains the specifications of calcium chloride, including the product number (Prod. No.), CAS number, supplier information, and relevant physicochemical properties obtained from the manufacturers' technical data sheets. See DOI: https://doi.org/10.1039/d6ra04235j.
