# Supplementary material for: Alginate Micro-fibers encapsulated with oregano essential oil with improved antibacterial efficacy
Source: RSC Adv. 2026 Jul 17;16(38):41483–97. doi: 10.1039/d6ra04235j (PMC13378664; doi:10.1039/d6ra04235j)
Supplement: Supplementary file 1 [file RA-016-D6RA04235J-s001.pdf]

Supplementary Information (SI) for RSC Advances.
This journal is © The Royal Society of Chemistry 2026

Alginate Micro-fibers encapsulated with Oregano Essential Oil with improved Antibacterial Efficacy

Supporting Information

Table S1. The Product Specification of the Sodium Alginate (SAlgP) (Product Number: **W201502**, CAS Number: **9005-38-3**, MDL: **MFCD00081310,** was obtained from Sigma's official website: www.sigmaaldrich.com

TEST	Specification
Appearance (Color)	White to Beige and Faint Brown to Light Brown
Appearance (Form)	Solid
Infrared spectrum	Conforms to Structure
Loss on Drying	≤15.5 %
Viscosity	5.0 - 40.0 cps
c = 1%, Water @ 25°C	5 - 8
pH	
c = 1%, Water @ 25°C Arsenic (As)	≤ 3 ppm
Cadmium (Cd)	≤ 1 ppm
Mercury (Hg)	≤ 1 ppm
Lead (Pb)	≤ 10 ppm

Specification PRD.4. ZQ5.10000036587

Table S2. The Product Specification of the Calcium Chloride ($Cacl_2$) (Product Number: **C1016**, CAS Number: **10043-52-4**, MDL: **MFCD00010903,** was obtained from Sigma's official website: www.sigmaaldrich.com

TEST	Specification
Appearance (Color)	White
Appearance (Form)	Granules
Solubility (Color)	Colorless
Solubility (Turbidity)	Clear to Hazy
Formula Weight	110.98 g/mol
Anhydrous,	≤7.0mm
Granular	≥93.0%
100 mg/mL H_2O, which becomes clear to very slightly hazy after addition of 1 drop of HCl	
Atomic Emission by ICP (Ca) Positive for Calcium	Positive
Purity Titration by EDTA	≥ 93.0 %
Particle Size <= 7.0 mm	Pass

Specification: PRD.1.ZQ5.10000017179
